# Better dual-task processing in simultaneous interpreters

**DOI:** 10.3389/fpsyg.2015.01590

**Published:** 2015-10-14

**Authors:** Tilo Strobach, Maxi Becker, Torsten Schubert, Simone Kühn

**Affiliations:** ^1^Medical School HamburgHamburg, Germany; ^2^Humboldt University of BerlinBerlin, Germany; ^3^University Medical Center Hamburg–EppendorfHamburg, Germany; ^4^Max Planck Institute for Human DevelopmentBerlin, Germany

**Keywords:** simultaneous interpreting, dual tasks, task coordination, executive functions

## Abstract

Simultaneous interpreting (SI) is a highly complex activity and requires the performance and coordination of multiple, simultaneous tasks: analysis and understanding of the discourse in a first language, reformulating linguistic material, storing of intermediate processing steps, and language production in a second language among others. It is, however, an open issue whether persons with experience in SI possess superior skills in coordination of multiple tasks and whether they are able to transfer these skills to lab-based dual-task situations. Within the present study, we set out to explore whether interpreting experience is associated with related higher-order executive functioning in the context of dual-task situations of the Psychological Refractory Period (PRP) type. In this PRP situation, we found faster reactions times in participants with experience in simultaneous interpretation in contrast to control participants without such experience. Thus, simultaneous interpreters possess superior skills in coordination of multiple tasks in lab-based dual-task situations.

## Introduction

When individuals experience a prolonged mismatch between their functional organismic supplies and environmental demands, the plastic character of the human cognitive system adapts its functioning to such demands (e.g., Lövdén et al., 2010). For example, extensive experience in playing action video games with their multiple and complex tasks optimizes the ability to perform and coordinate two simultaneous tasks in a dual-task paradigm (e.g., Strobach et al., 2012a). For the case of experience in professional interpreting with transfer from auditory input to verbal output in a first and a second language, respectively, there is ample evidence showing that this activity is associated with better working memory performance which thus points to working memory processes as critical for interpreting (e.g., Darò and Fabbro, 1994; Christoffels et al., 2003, 2006; Liu et al., 2004; Padilla et al., 2005; Tzou et al., 2011). This association is plausible given that activities such as reformulating linguistic material from a first to a second language requires holding intermediate processing steps in working memory. While there is evidence in the literature that superior processing of concurrent working memory content is associated with experience in interpreting (Padilla et al., 2005), it is, however, an open issue whether persons with experience in interpreting possess superior skills in the performance and coordination of multiple tasks in dual-task situations. In particular, within the present study, we set out to explore whether interpreting experience is associated with dual-task related executive functioning (e.g., Baddeley, 1986; Miyake et al., 2000; Miyake and Friedman, 2012).

We hypothesize that dual-task coordination skills are important for persons with interpreting experience since interpreting is highly complex and requires the performance and coordination of multiple, simultaneous tasks: analysis and understanding of the discourse in a first language, reformulating linguistic material, storing of intermediate processing steps, language production, etc. (Gile, 1997; Padilla et al., 2005). Online coordination of simultaneous tasks is a particularly relevant skill for simultaneous interpreting (SI), which is characterized by simultaneous listening and speaking. Alternative types of interpreting such as consecutive interpreting (CI) require multiple task coordination to a lesser degree; in fact, this interpreting type particularly requires a non-simultaneous, but sequential alternation between listening and speaking (Christoffels and de Groot, 2009).

A recent study (Morales et al., 2015) approached the issue of multiple task coordination when investigating performance in a single and dual *n*-back updating task (Jaeggi et al., 2008) in persons with SI experience (SIs) and controls not involved with any interpreting activity (no SIs). The results of both the single and dual updating conditions (i.e., one *n*-back task vs. two simultaneous *n*-back tasks) demonstrated generally better performance in SIs, showing their superior performance in a task requiring monitoring and updating of information. However, findings of a general advantage in SIs vs. controls across the single and dual-task conditions provide no evidence that is consistent with the assumption of improved multiple task coordination with SI experience. This improvement requires a specific advantage for the dual-updating condition (see below). General SI advantages such as the ones demonstrated by Morales et al. (2015) could not be specifically attributed to multiple task coordination, but to a set of general mechanisms (e.g., advanced baseline task processing abilities as measured with a single-task advantage in SI). The lacking evidence for multiple task coordination, however, may result from the applied type of test of multiple tasks. That is, *n*-back tasks do not instruct for speeded responses, but for accuracy, which may not provide a sensitive condition to precisely control the measurement of task processing and thus the analysis of multiple task coordination processes (Strobach et al., 2015a). A similar argument is warranted for a task situation in a study of Padilla et al. (2005, Experiment 2) who combined word list learning while performing a visual object tracking task. Similar to the findings of Morales et al. (2015), there was no conclusive evidence for advanced multiple task coordination in this study.

**FIGURE 1:**
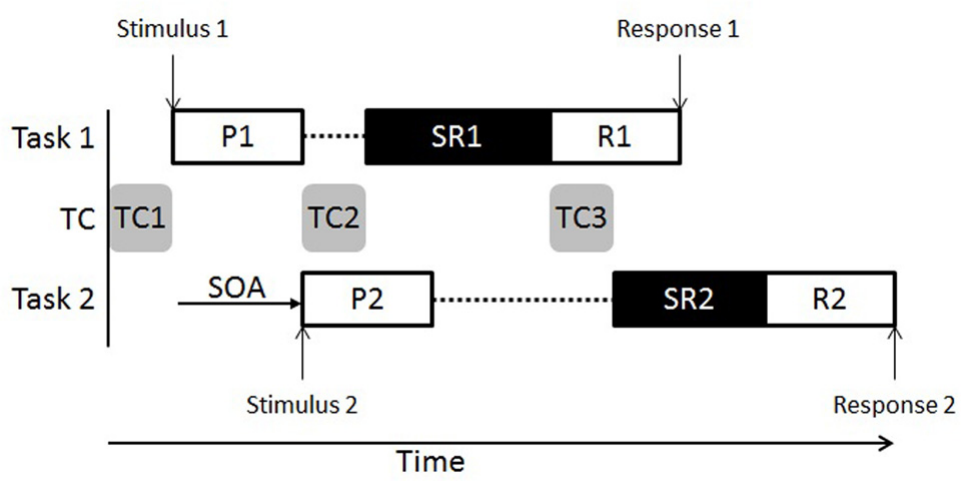
**Illustration of Task 1 and Task 2 processing in dual tasks of the Psychological Refractory Period type.** While stimulus processing and response execution can be performed in parallel with any other processes, processes of task coordination (TC) and processing of stimulus–response mapping cannot be processed with each other, but only sequentially. P1 and P2, perception stages; SR1 and SR2, stimulus-response mapping stages; R1, R2, response stages; TC1, TC2, and TC3, task coordination stages; SOA, stimulus onset asynchrony.

One prominent paradigm to test the skill to perform and coordinate simultaneous tasks with a strictly timed protocol is the dual-task situation of the psychological refractory period (PRP) type (e.g., Welford, 1952; Pashler, 1994; Schubert, 1999, 2008; Logan and Gordon, 2001; Strobach et al., 2015a). In this PRP dual-task situation, participants perform speeded responses on stimuli of a first and a second choice reaction time (RT) task (Task 1 vs. Task 2). A variable interval separates stimulus presentations of Task 1 and Task 2 (i.e., stimulus onset asynchrony, SOA) and participants are instructed to give priority to the performance of Task 1. This specific PRP situation typically shows dual-task performance costs by RT in Task 2 (RT2) that increase with decreasing SOA (i.e., the so-called PRP effect; RTs in Task 1 (RT1) are typically constant and independent of SOA. The PRP effect can be explained with a capacity-limited processing bottleneck at which certain stages of the component tasks cannot be processed simultaneously, but sequentially. In choice RT component task, a promising bottleneck candidate is the central stimulus–response mapping stage. This task stage is related to the selection of response information based on incoming stimulus information and is located after a perception and before a motor stage (Pashler, 1994; Schubert, 1999; **Figure 1**). In the context of a PRP dual task, the stimulus–response mapping stage in Task 2 has to wait until this stage in Task 1 has left the bottleneck, explaining the PRP effect.

In a PRP situation, executive functions are particularly required to efficiently regulate and coordinate two concurrent task processing streams (Kramer et al., 1995; Meyer and Kieras, 1997; Miyake et al., 2000; Miyake and Friedman, 2012). In addition to capacity limitations within the component tasks (e.g., at the stimulus–response mapping stage), these functions also delay dual-task processing and contribute to dual-task performance costs. In the literature, several specific task coordination components were illustrated. Particularly, task coordination could be required to efficiently instantiate and activate relevant task information in working memory before the beginning of Task 1 processing (task coordination 1 [TC1] in **Figure 1**; e.g., de Jong, 1995; Hartley and Little, 1999; Stelzel et al., 2009; Strobach et al., 2014). It could also be associated with coordinating the access of the stimulus–response mapping stages of the individual component tasks to the bottleneck mechanism (TC2 in **Figure 1**; e.g., Szameitat et al., 2002, 2006; Sigman and Dehaene, 2006; Hendrich et al., 2012; Strobach et al., 2015b). Furthermore, task coordination could be related to a switch and reactivation of relevant task information after stimulus–response mapping of the component task that completed the bottleneck mechanism first and before this mapping of the component task that entered this mechanism second (TC3 in **Figure 1**; e.g., Lien et al., 2003; Maquestiaux et al., 2004; Liepelt et al., 2011; Strobach et al., 2012b). Recent studies have demonstrated that dual-task performance can be improved with dual-task practice (Ruthruff et al., 2006; Kamienkowski et al., 2011; Strobach et al., 2012c,d), which is, among other mechanisms, a result of the acquisition of optimized task coordination skills specifically located at the bottleneck switch (i.e., TC3). In contrast, there is no such skill acquisition seen in response to single-task practice that does not require task coordination (Liepelt et al., 2011; Strobach et al., 2012b, 2015c). Importantly, there is evidence for the acquisition of task coordination skills in dual tasks that are not specific for the practiced situation but can be transferred to new task contexts (e.g., Kramer et al., 1995; Strobach et al., 2012b; Anguera et al., 2013).

Given this practice-related plasticity and transferability of task coordination skills as well as the plausible application of such skills in SI in contrast with controls not experienced in SI (however, experienced in alternative interpreting styles such as CI), we predict that dual-task performance is better in participants with SI experience in contrast to control participants in a PRP situation combining an auditory Task 1 and visual Task 2 (Salminen et al., 2012). However, the locus of the potential dual-task performance advantage in participants with SI experience specifies the focus of improved task coordination skills. If there is an advantage in Task 1 and Task 2 performance, this could be attributed to better task instantiation at the beginning of a dual-task trial (TC1, **Figure 1**) and/or bottleneck access coordination (TC2). In contrast, if performance advantage is limited to Task 2, this would indicate a better switch and reactivation of relevant task information between the bottleneck stages (TC3).

## Materials and Methods

### Participants

Fifty-three interpreters and translators were recruited via the BDÜ (Federal Association of Interpreters and Translators) mailing list for the present study. Our recruitment letter made clear that we were equally interested in both occupational groups (i.e., interpreters and translators) to avoid motivational differences (Schubert and Strobach, 2012; Green et al., 2014). According to their self-reported experience, this group was separated into subgroups with experience in (1) SI and (2) control participants (no SI experience, but experience in CI and translating). The data of four participants were not included into the final data set, because of problems with data recording (two participants) and refusing to perform the present task situation (two participants). These drop-outs left 49 participants for the final data set (36 female; age [in years]: *Mean* [*M*] = 42.9, *SD* = 11.2, *range* = 26–69): 27 SIs and 22 no SIs. The remaining biographical details and details of these subgroups’ interpreting experience are listed in **Table 1**. All participants were also screened for normal or corrected-to-normal vision and hearing based on self-report. Approval by an ethics committee was obtained before commencement of the study, which was conducted in strict accordance with the ethics policies of the German Psychological Society (approval executed by TransMIT GmbH, Gießen, Germany). Written informed consent was obtained from each participant.

**Table 1:** Biographical details of participants with/ without simultaneous interpreting (SIs vs. no SIs) experience.

	SIs	No SIs	Difference (*t*-test)	
			*t*	*p*
N of participants	27	22		
N females	22	15		
Age (in years, mean [*SD*])	42.74 [11.76]	42.59 [10.74]	0.05	0.96
Native language (N and language)	18 German 9 Other	18 German 4 Other		
First foreign language (N and language)	14 English 9 German 4 Other	12 English 4 German 6 Other		
Age of acquisition of 1st foreign language (in year, mean [*SD*])	11.70 [4.89]	13.18 [7.94]	0.80	0.43
N with structured education in interpreting/translation (e.g., university education)	23	16		
Duration of structured education in interpreting/translating (in years, mean [*SD*])	4.27 [2.16]	5.44 [1.97]	1.70	0.10
N freelancers	25	20		
Working time spent on consecutive interpreting per week (in hours, mean [*SD*])	2.83 [4.87]	1.32 [3.51]	1.21	0.23
Duration of professional interpreting and/ or translating until time of testing (in years, mean [*SD*])	14.00 [10.75]	12.93 [10.51]	0.35	0.73

*SD, standard deviation.*

### Apparatus

Visual stimuli in the following experiment were presented on a Lenovo thinkpad laptop and auditory stimuli were presented via HP H2500 headphones connected to this laptop. The experiment was controlled by the software package Presentation.

### Design and Procedure

**FIGURE 2:**
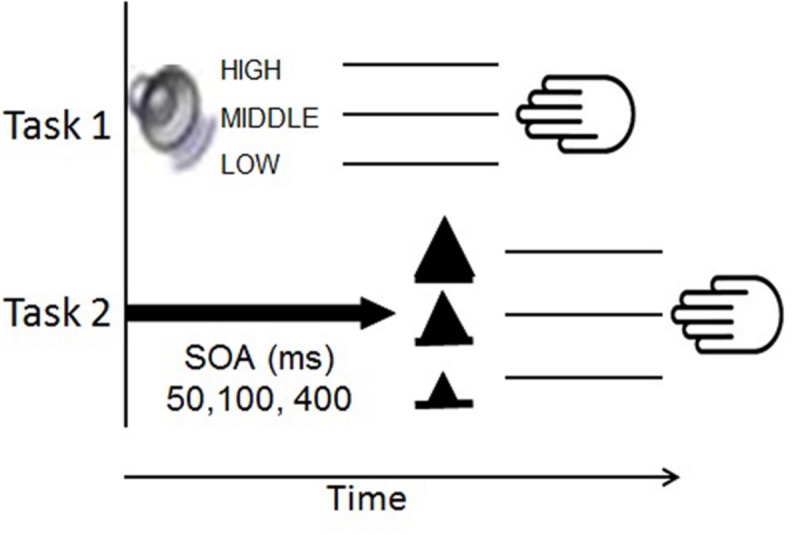
**Illustration of stimulus information, stimulus-response mappings rules, and response information of Task 1 and Task 2 in dual-task trials of the Psychological Refractory Period type.** SOA, stimulus onset asynchrony.

Participants performed an auditory and visual RT task in single-task and dual-task trials. We selected tasks with different input modalities (visual and auditory) to minimize possible interference between perceptual input processes (Wickens, 1989). In this way, we can ensure that differences in dual-task performance largely reflect specific differences in processes of executive control on selecting and executing multiple responses (e.g., Meyer and Kieras, 1997). In Task 1, we presented sine-wave tones with pitches of either 350 (low), 900 (middle), or 1,650 (high) Hz and participants responded with the index, middle, and ring finger of the right hand, respectively. In Task 2, we used small, middle, and large visually presented triangles, which required responses with the ring, the middle, and the index finger of the left hand, respectively (**Figure 2**). The triangles were white in front of a black background. This background color was also used in Task 1.

Participants performed single-task blocks in which only one of the two tasks was presented and performed at a time at a time. They also performed dual-task blocks that included the presentation of both tasks. Trials of single-task blocks started with the presentation of three white dashes next to each other of which the middle dash was located at the center of the screen. The dashes remained on screen until the end of each single-task trial. After 500 ms, an auditory stimulus (i.e., sine-wave tone) was presented for 50 ms in auditory single-task block trials, or a visual stimulus (i.e., triangle) appeared centrally in the visual single-task block trials until response or a time interval of 2,500 ms. Similar to single-task trials, dual-task trials (**Figure 2**) also started with the presentation of three white dashes, which remained on screen until the end of the trial. After 500 ms, an auditory stimulus was presented, followed by the presentation of a visual stimulus. The interval between the onsets of both stimuli (i.e., SOA) was 50, 100, or 400 ms.

Single-task blocks consisted of 54 single-task trials and stimuli were presented with the same frequency in random order. In the 54 trials of the dual-task blocks, auditory, and visual stimuli were presented with the same frequency and stimuli were selected randomly. The number of trials with SOAs of 50, 100, and 400 ms was balanced within blocks. Participants were instructed to respond as quickly and as accurately as possible in single-task blocks as well as in the dual-task blocks. In dual-task blocks, priority was further instructed for Task 1 (i.e., the auditory task). At the beginning of the experiment, one single-task block of Task 1 and one single-task block of Task 2 were conducted in this order. Following these two single-task blocks, three blocks consisting of dual tasks trials were conducted (in total: 162 dual-task trials). In this way, we realized an identical number of trials of each component task in single tasks and at the individual SOA levels in dual tasks (i.e., 54 single-task trials as well as 54 dual-task trials with SOA of 50, 100, and 400 ms).

## Results

The single-task and PRP dual-task situations resulted in behavioral data consisting of RTs and error rates for Task 2 and Task 1 (we start with Task 2 presentation, because of the presence of prominent PRP effect in this task). In single-task trials, we analyzed the resulting four measures (i.e., Task 2 RTs/error rates, Task 1 RTs/error rates) in the following model: mixed-measures ANCOVA with the between-subjects factor SI (SIs vs. no SIs), and the covariates Amount of CI experience (number of hours per week), Amount of structured education (in interpreting/ translating, in years) as well as Age (in years); particularly, the latter aspects are relevant to control for the proven effects of aging (e.g., Verhaeghen et al., 2003; Verhaeghen, 2011; Strobach et al., 2015d) and formal education (Vallesi, 2015) on dual-task performance. In PRP dual tasks, we added the within-subjects factor SOA (50, 100, 400 ms) to this model. Since error rates are typically less sensitive to demonstrate group differences in the PRP paradigm (Strobach et al., 2015a), superior dual-task performance in SIs would be primarily indicated by their faster RTs in this model.

Furthermore, we were interested whether this potential superiority is related to processing the component task stages or to task coordination processes. An exclusive superiority in dual-task performance in contrast to single-task performance would be indicative for better task coordination, while no such exclusive superiority would not point to this better executive processing, but to differences in baseline processing speed, for instance. As a consequence, we compared dual-task with single-task performance in Task 1 and Task 2. In fact, we focused on interactions with the critical factor SI and Task condition (single task vs. dual task) when we analyzed the single-task data and the dual-task data of the 50-ms SOA condition. This latter SOA condition provides the largest amount of temporal overlap when processing two tasks (Pashler and Johnston, 1989). Therefore, this condition represents the most demanding dual-task condition and was regarded the most solid test for task coordination skills (Strobach et al., 2012a).

The combined analysis of Task 1 vs. Task 2 also focused on the group factor SI and its potential interactions with SOA and Task (Task 1 vs. Task 2) when comparing these tasks’ dual-task performance levels. This combined analysis was conducted to investigate whether potential differences between the groups’ dual-task performance are specific for either component task (i.e., Task 1 or Task 2). In general, we excluded trials with erroneous and/or omitted responses in one or both component tasks from all RT analyses.

### Task 2

#### Dual-task RTs

**FIGURE 3:**
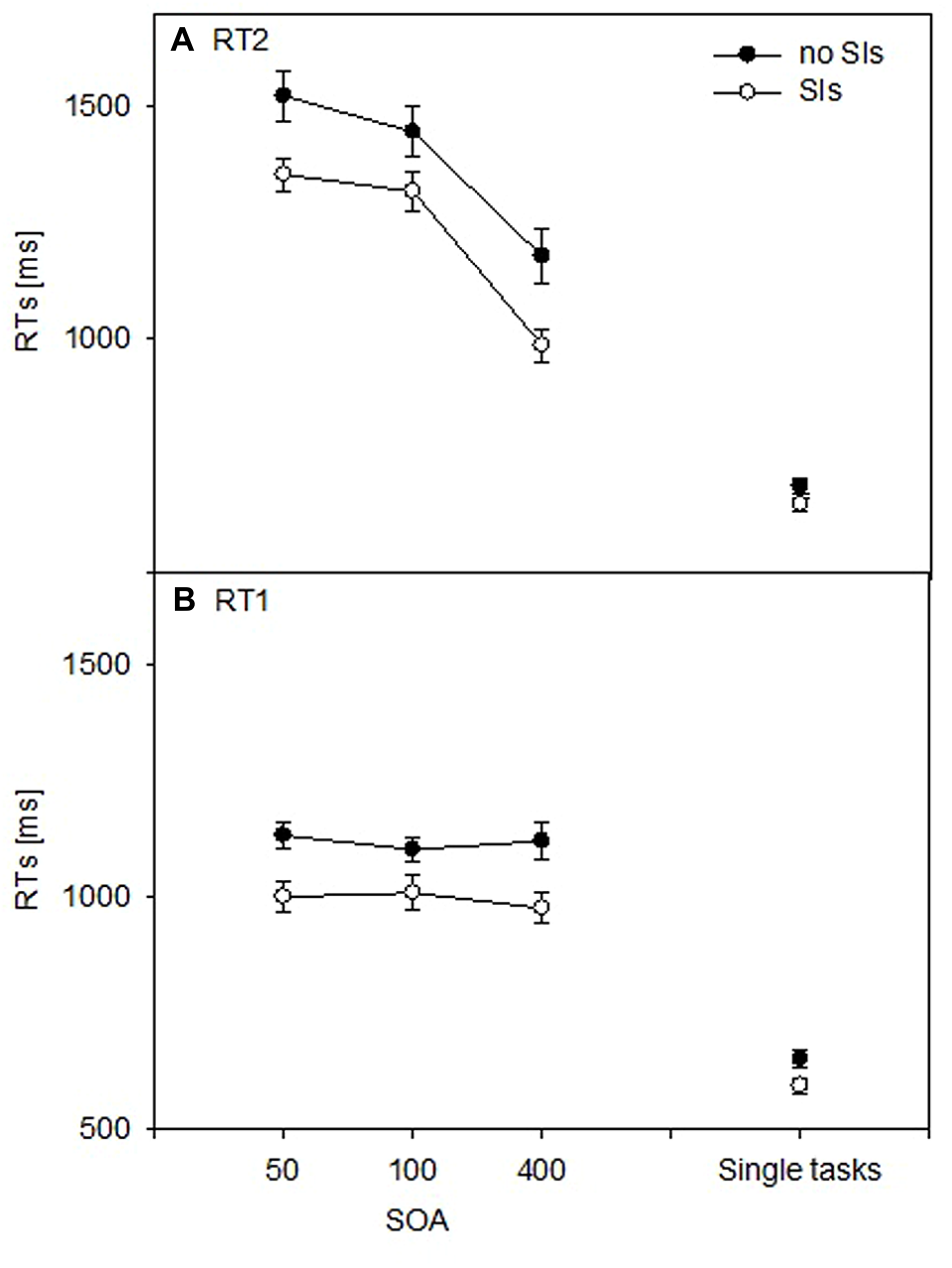
**Mean reaction times (RTs) in participants with experience in simultaneous interpreting (SIs) and without such experience (no SIs), dual tasks (SOA: 50, 100, and 400 ms) and single tasks. (A)** Task 2. **(B)** Task 1.

The RT data of Task 2 (**Figure 3A**) demonstrated the typical PRP effect (e.g., Pashler, 1994; Schubert, 1999), *F*(2,88) = 34.223, *p* < 0.001, $\eta_{p}^{2}$ = 0.44, and increasing RTs with decreasing SOAs. Importantly, the factor SI was significant, *F*(1,44) = 8.987, *p* < 0.01, $\eta_{p}^{2}$ = 0.17. This effect indicated that participants with SI experience were generally faster (*M* = 1,218 ms) than participants without SI experience (*M* = 1,385 ms). This dual-task advantage was not modulated since no covariate or interaction was significant [Amount of CI experience: *F*(1,44) < 1; Age: *F*(1,44) < 1; Amount of structured education: *F*(1,44) = 2.974, *p* = 0.10; SOA × SI: *F*(2,88) = 1.745, *p* = 0.18; SOA × Age: *F*(2,88) < 1; SOA × Amount of CI experience: *F*(2,88) < 1], except the combination of SOA × Amount of structured education, *F*(2,88) = 5.687, *p* < 0.01, $\eta_{p}^{2}$ = 0.11; the latter points to the influence of education (in particular, structured education in interpreting/translating) on dual-task performance (see also Vallesi, 2015).

#### Single-task RTs

As illustrated in **Figure 3A**, there was no significant main effect or covariate [SI: *F*(1,44) = 1.893, *p* = 0.18; Amount of CI experience: *F*(1,44) < 1; Age: *F*(1,44) = 1.022, *p* = 0.32; Amount of structured education: *F*(1,44) < 1].

#### Single and Dual-task RTs

The interaction of Task condition and SI was significant, *F*(1,44) = 8.984, *p* < 0.01, $\eta_{p}^{2}$ = 0.17. This interaction indicated similar single-task RTs in participants with SI experience vs. participants without such experience, *t*(47) = 1.614, *p* > 0.11, while there were significantly faster RTs at the 50-ms SOA level in SI-experienced participants, *t*(47) = 2.690, *p* < 0.01. In addition, there were generally faster RTs in SI-experienced participants in contrast to participants with no such experience, *F*(1,44) = 6.971, *p* < 0.05, $\eta_{p}^{2}$ = 0.14, as well as in single tasks in contrast to dual tasks, *F*(1,44) = 292.361, *p* < 0.001, $\eta_{p}^{2}$ = 0.87. No other covariate and interaction was significant [Amount of CI experience: *F*(1,44) < 1; Age: *F*(1,44) < 1; Amount of structured education: *F*(1,44) = 2.193, *p* = 0.15; Task condition × Amount of CI experience: *F*(1,44) < 1; Task condition × Age: *F*(1,44) < 1; Task condition × Amount of structured education: *F*(1,44) = 3.363, *p* = 0.08].

#### Dual-task Error Rates

Task 2’s error analysis showed an effect of SI, *F*(1,44) = 4.974, *p* < 0.05, $\eta_{p}^{2}$ = 0.10, with lower error rates in persons with SI experience (*M* = 6.5%) in comparison to person without such experience (*M* = 12.7%). This finding is consistent with the SI advantage in the RT data. No other main effect, covariate or interaction was significant [**Table 2**; SOA: *F*(2,88) < 1; Amount of CI experience: *F*(1,44) < 1; Age: *F*(1,44) < 1; Amount of structured education: *F*(1,44) = 3.327, *p* = 0.09; SOA × SI: *F*(2,88) < 1; SOA × Amount of CI experience: *F*(2,88) < 1; SOA × Age: *F*(2,88) < 1]. Thus, advantages in dual-task processing speed in participants with SI experience are not a result of a speed-accuracy trade-off.

**Table 2:** Mean error rates (in brackets: standard errors) in Task 1 and Task 2, participants with experience in simultaneous interpreting (SIs) and no such experience (no SIs), dual tasks (Stimulus onset asynchrony, SOA: 50, 100, 400) and single tasks.

		SOA			
		50	100	400	Single tasks
No SIs	Task 2	0.13 (0.03)	0.13 (0.03)	0.13 (0.03)	0.05 (0.01)
	Task 1	0.17 (0.03)	0.14 (0.03)	0.11 (0.03)	0.09 (0.02)
SIs	Task 2	0.06 (0.01)	0.07 (0.01)	0.06 (0.01)	0.04 (0.01)
	Task 1	0.10 (0.02)	0.10 (0.02)	0.08 (0.02)	0.09 (0.01)

#### Single-task Error Rates

No main effect, covariate or interaction was significant [**Table 2**; SI: *F*(1,44) < 1; Amount of CI experience: *F*(1,44) < 1; Age: *F*(1,44) < 1; Amount of structured education: *F*(1,44) = 2.078, *p* = 0.16].

#### Single and Dual-task Error Rates

The comparison of single and dual-task error performance showed lower error rates in the former in contrast to the latter condition, *F*(1,44) = 7.447, *p* < 0.01, $\eta_{p}^{2}$ = 0.15. Further, persons with SI experience generally showed lower error rates than persons without such experience, *F*(1,44) = 4.749, *p* < 0.05, $\eta_{p}^{2}$ = 0.10. No other main effect, covariate or interaction was significant [Amount of CI experience: *F*(1,44) < 1; Age: *F*(1,44) < 1; Amount of structured education: *F*(1,44) = 3.665, *p* = 0.08; Task condition × SI: *F*(1,44) = 2.601, *p* = 0.11; Task condition × Amount of CI experience: *F*(1,44) < 1; Task condition × Age: *F*(1,44) < 1; Task condition × Amount of structured education: *F*(1,44) = 3.100, *p* = 0.09).

### Task 1

#### Dual-task RTs

As illustrated in **Figure 3B**, the analysis of Task 1 RTs revealed a main effect of SI, *F*(1,44) = 5.062, *p* < 0.05, $\eta_{p}^{2}$ = 0.10. This main effect indicated generally faster RTs in participants experienced in SI (*M* = 1011 ms) in contrast to participants with no such experience (*M* = 1125 ms). Further, there was influence of the covariate Amount of structured education, *F*(1,44) = 4.488, *p* < 0.05, $\eta_{p}^{2}$ = 0.09, and an interaction of this covariate and SOA, *F*(2,88) = 5.854, *p* < 0.01, $\eta_{p}^{2}$ = 0.12; no other main effect, covariate or interaction was significant [SOA: *F*(2,88) = 2.670, *p* = 0.08; Amount of CI experience: *F*(1,44) < 1; Age: *F*(1,44) = 1.151, *p* = 0.29; SOA × SI: *F*(2,88) = 2.347, *p* = 0.10; SOA × Amount of CI experience: *F*(2,88) < 1; SOA × Age: *F*(2,88) = 1.221, *p* = 0.30].

#### Single-task RTs

There was a main effect of SI, *F*(1,44) = 5.991, *p* < 0.05, $\eta_{p}^{2}$ = 0.12 (**Figure 3B**), demonstrating faster RTs in SIs (*M* = 595 ms) than in no SIs (*M* = 652 ms). The covariates were non-significant [Amount of CI experience: *F*(1,44) < 1; Age: *F*(1,44) < 1; Amount of structured education: *F*(1,44) = 3.349, *p* = 0.08].

#### Single and Dual-task RTs

The interaction of Task condition and SI was significant, *F*(1,44) = 5.385, *p* < 0.05, $\eta_{p}^{2}$ = 0.12. This finding indicated a smaller RT difference between SIs and no SIs in single tasks (*M* = 58 ms) than in dual tasks (*M* = 112 ms). Furthermore, there were generally faster RTs in SI-experienced participants in contrast to participants with no such experience, *F*(1,44) = 8.148, *p* < 0.01, $\eta_{p}^{2}$ = 0.16, as well as in single tasks in contrast to dual tasks, *F*(1,44) = 84.020, *p* < 0.001, $\eta_{p}^{2}$ = 0.66. No other covariate or interaction was significant [Amount of CI experience: *F*(1,44) < 1; Age: *F*(1,44) < 1; Amount of structured education: *F*(1,44) = 2.313, *p* = 0.10; Task condition × Amount of CI experience: *F*(1,44) < 1; Task condition × Age: *F*(1,44) = 2.552, *p* = 0.12; Task condition × Amount of structured education: *F*(1,44) = 1.421, *p* = 0.24].

#### Dual-task Error Rates

The analysis of error rates in Task 1 revealed an influence of the amount of structured education, *F*(1,44) = 4.098, *p* < 0.05, $\eta_{p}^{2}$ = 0.09. There were no other main effects, significant covariates or interactions [**Table 2**; SI: *F*(1,44) = 3.315, *p* = 0.08; Amount of CI experience: *F*(1,44) < 1; Age: *F*(1,44) < 1; SOA × SI: *F*(2,88) = 2.272, *p* = 0.11; SOA × Amount of CI experience: *F*(2,88) < 1; SOA × Age: *F*(2,88) < 1; SOA × Amount of structured education: *F*(2,88) < 1].

#### Single-task Error Rates

No main effect or covariate was significant [**Table 2**; SI: *F*(1,44) < 1; Amount of CI experience: *F*(1,44) < 1; Age: *F*(1,44) < 1; Amount of structured education: *F*(1,44) = 1.772, *p* = 0.19].

#### Single and Dual-task Error Rates

The comparison of single and dual-task error performance showed lower error rates in the former in contrast to the latter condition, *F*(1,44) = 4.949, *p* < 0.05, $\eta_{p}^{2}$ = 0.10. Furthermore, we found lower error rates in SI-experienced participants vs. participants with no such experience under dual-task conditions, while there were similar error rates in single tasks, *F*(1,44) = 5.347, *p* < 0.05, $\eta_{p}^{2}$ = 0.11, supporting the assumption of a dual-task advantage in SIs. No other main effect, covariate or interaction was significant [SI: *F*(1,44) = 3.464, *p* = 0.07; Amount of CI experience: *F*(1,44) < 1; Age: *F*(1,44) < 1; Amount of structured education: *F*(1,44) = 2.884, *p* = 0.10; Task condition × Amount of CI experience: *F*(1,44) < 1; Task condition × Age: *F*(1,44) < 1; Task condition × Amount of structured education: *F*(1,44) < 1].

### Task 2 and Task 1

#### Dual-task RTs

This analysis showed a main effect of SI, *F*(1,44) = 7.128, *p* < 0.05, $\eta_{p}^{2}$ = 0.14, demonstrating generally faster RTs in SIs in contrast to no SIs. However, there was no interaction including the factor SI [Task: *F*(1,44) = 56.043, *p* < 0.001, $\eta_{p}^{2}$ = 0.56; SOA: *F*(2,88) = 9.769, *p* < 0.001, $\eta_{p}^{2}$ = 0.18; Amount of CI experience: *F*(1,44) < 1; Age: *F*(1,44) < 1; Amount of structured education: *F*(1,44) = 4.256, *p* < 0.05, $\eta_{p}^{2}$ = 0.09; Task × SOA: *F*(2,88) = 506.675, *p* < 0.001, $\eta_{p}^{2}$ = 0.92; Task × SI: *F*(1,44) = 2.573, *p* = 0.08; Task × Amount of CI experience: *F*(1,44) < 1; Task × Age: *F*(1,44) = 2.211, *p* < 0.14; Task × Amount of structured education: *F*(1,44) < 1; SOA × SI: *F*(2,88) = 2.573, *p* = 0.08; SOA × Amount of CI experience: *F*(2,88) < 1; SOA × Age: *F*(2,88) = 1.092, *p* = 0.34; SOA × Amount of structured education: *F*(2,88) = 6.032, *p* < 0.01, $\eta_{p}^{2}$ = 0.12; Task × SOA × SI: *F*(2,88) < 1; Task × SOA × Amount of CI experience: *F*(2,88) = 2.908, *p* = 0.06; Task × SOA × Age: *F*(2,88) < 1; Task × SOA × Amount of structured education: *F*(2,88) < 1]. Thus, there is no evidence that SI-related performance variations in Task 2 are independent from those variations in Task 1.

#### Dual-task Error Rates

There was no significant main effect of or interaction with SI [SI: *F*(1,44) = 3.416, *p* = 0.07; Task: *F*(1,44) < 1; SOA: *F*(2,88) < 1; Amount of CI experience: *F*(1,44) < 1; Age: *F*(1,44) < 1; Amount of structured education: *F*(1,44) < 1; SI × Task: *F*(1,44) < 1; Task × SOA: *F*(2,88) = 1.325, *p* = 0.27; Task × Amount of CI experience: *F*(1,44) < 1; Task × Age: *F*(1,44) < 1; Task × Amount of structured education: *F*(1,44) < 1; SOA × SI: *F*(2,88) < 1; SOA × Amount of CI experience: *F*(2,88) = 1.186, *p* = 0.31; SOA × Age: *F*(2,88) < 1; SOA × Amount of structured education: *F*(2,88) < 1; Task × SOA × SI: *F*(2,88) = 2.175, *p* = 0.12; Task × SOA × Amount of CI experience: *F*(2,88) < 1; Task × SOA × Age: *F*(2,88) < 1; Task × SOA × Amount of structured education: *F*(2,88) < 1].

## Discussion

The present data provide evidence for the assumption of superior processing of dual tasks in participants with SI experience. In detail, RT2 and RT1 in dual tasks were faster in this type of participants in contrast to participants without SI experience (i.e., no SIs). These faster RTs extend single-task advantages of SIs, demonstrating that there is no general difference in baseline processing speed, but that the advantage observed is rather dual-task specific (Strobach et al., 2012b). This specific advantage points to superior task coordination in dual tasks in SIs in contrast to no SIs.

Thus, our data generally demonstrated superior executive functioning in SIs (e.g., Meyer and Kieras, 1997; Miyake et al., 2000). In detail, the faster dual-task RTs in Task 1 in participants experienced in SI compared to participants without experience in SI suggest task-coordination skills for an optimized activation of relevant task information (TC1 in **Figure 1**; e.g., de Jong, 1995; Strobach et al., 2014). Furthermore, this Task 1 advantage in SI-experienced participants may also point to optimized coordination of bottleneck access (TC2 in **Figure 1**; e.g., Sigman and Dehaene, 2006; Hendrich et al., 2012). These optimized task coordination processes (i.e., activation of relevant task information and bottleneck access coordination) are the essential components of task coordination skills to explain the difference between participants with SI experience vs. without experience in SI. However, there is no evidence for additionally optimized task coordination skills in SI-experienced participants such as a better regulation of bottleneck access (TC3 in **Figure 1**; e.g., Lien et al., 2003; Strobach et al., 2012b). Such evidence would stem from the combined RT analysis of Task 1 and Task 2 showing that group differences in RT2 extend those differences in RT1. However, our combined analysis (see RESULTS section) indicated no such result pattern. Thus, the most parsimonious explanation is that activation of task-relevant information (plus coordination of bottleneck access) is the essential task-coordination process that is superior in SIs.

In the present study, the superior coordination is demonstrated in a situation that combines non-verbal tasks and is structurally dissimilar from interpreting. This finding is consistent with the assumption that characteristics of interpreters are demonstrated in a far-transfer situation (Karbach and Kray, 2009) and is consistent with studies showing the transfer after lab-based dual-task training (e.g., Strobach et al., 2012a,b, 2014). In general, our conclusions extend the findings of previous studies that investigated characteristics of SIs in less-controllable situations with multiple tasks including working memory tasks (Padilla et al., 2005; Morales et al., 2015). The present task situation was able to provide conclusive evidence for superior multiple task coordination skills and specify the location of these skills in a dual-task processing architecture. Furthermore, our findings of superior multiple task coordination complement findings of other studies with advanced working memory abilities in SIs (e.g., Christoffels et al., 2003, 2006; Padilla et al., 2005; Tzou et al., 2011). In fact, such advanced working memory abilities might explain superior executive functioning and task coordination skills, since, among others, superior task coordination involves the optimized activation of relevant task information in working memory (e.g., Strobach et al., 2014). Unfortunately, we performed no working memory test (e.g., on working memory updating or capacity) in the present study to directly investigate this assumption or alternative assumptions about the impact of basic cognitive skills (i.e., intelligence) on task coordination.

At this point, we would like to stress, that we do not claim that interpreting is the source for advantages in SIs vs. participants with no SI-experience. Rather, we provide data that specify characteristics of SI participants. Such characteristics might be essential components in the complex process of SI constituting a combination of tasks like analysis and understanding of the discourse in a first language, transfer from a first to a second language, language production, etc., in association with other skills such as optimized working memory processing (e.g., Padilla et al., 2005). Thus, in general, we speculate that superior task coordination in the present PRP situation might be associated with the performance and coordination of multiple, simultaneous tasks during interpreting (Miyake and Friedman, 2012).

Superior task coordination skills and thus executive control in SIs is consistent with studies showing that bilingualism – the ability to understand, fluently speak, and frequently use two languages – is associated with cognitive benefits (Mägiste, 1980; Bialystok, 2001; Poulin-Dubois et al., 2013). In particular, executive functioning like mental flexibility, task switching, attentional and inhibitory control are assumed to be enhanced in bilinguals compared to monolinguals (Bialystok and DePape, 2009; Prior and MacWhinney, 2010; Gold et al., 2013). Similar to the present study with evidence for superior dual-task performance and task coordination skills in SIs, enhanced executive processing in bilinguals may be related to the specific demands to understand, fluently speak, and frequently use two languages. The reason why there is a stronger need to manage two languages is because recent research suggests that in fluent bilinguals, both language systems are not independently represented and uniquely accessed but both languages are activated at all times with interactions between them even when the context demands the use of just one language (Meuter and Allport, 1999).

Similar to other studies on the characteristics of interpreters, there are some critical methodological issues in the present study. For instance, in this study, the group of participants with experience in SI is not homogeneous (Gile, 1991; Massaro and Shlesinger, 1997; Christoffels and de Groot, 2009). That is, we have interpreters with a selection of different foreign languages, different education schedules, a large age range, etc. Furthermore, although the present findings are consistent with the general assumption that the cognitive system is plastic in a way that basic cognitive processes adapt to prolonged demands we cannot exclude that the observed group differences may be due to *a priori* differences that lead people to become SIs. Future studies should collect longitudinal data following interpreters over their course of education to determine whether SI challenges cause an optimization of dual-task coordination skills. In addition, future studies should realize alternative dual-task situations to specify the present findings of SIs’ advanced task coordination of simultaneous tasks.

## Conflict of Interest Statement

The authors declare that the research was conducted in the absence of any commercial or financial relationships that could be construed as a potential conflict of interest.
